# Sex differences in atrial fibrillation recurrence after catheter ablation: research status and progress

**DOI:** 10.1186/s13293-026-00914-9

**Published:** 2026-05-03

**Authors:** Yongzhi Cao, Hao Wang, Chengyu Shi, Yunfei Gu

**Affiliations:** 1 Department of Cardiology, Luoyang Central Hospital Affiliated to Zhengzhou University; Luoyang Key Laboratory of Myocardial Injury and Repair, Luoyang, Henan Province China; 2 Graduate School of Medicine, Dentistry and Pharmaceutical Sciences, Okayama University, Okayama, Japan

**Keywords:** Atrial fibrillation, Catheter ablation, Recurrence risk, Sex differences, Cardiac remodeling

## Abstract

Sex differences in the risk of recurrence after catheter (CA) ablation for atrial fibrillation (AF) have become a focal point of interest in recent years. This narrative review synthesizes the available evidence and, based on published reports, suggests that female patients may have a higher overall risk of postoperative recurrence than male patients, although substantial heterogeneity exists across studies. This is a narrative review and did not follow the methodological framework of a systematic review (e.g., PRISMA diagram, risk-of-bias assessment, and quantitative synthesis). Sex differences are not driven by a single biological factor but arise from the complex interaction of multidimensional factors, including hormonal levels, electrophysiological characteristics, cardiac remodeling, and clinical management. Current research remains constrained by methodological heterogeneity, inconsistent endpoint definitions, and inadequate adjustment for confounders, which undermine the robustness of the conclusions. Future efforts should advance rigorously designed prospective studies to test current hypotheses. On this basis, the development of sex-sensitive, individualized management pathways—encompassing precise preoperative assessment, substrate-guided ablation strategies, and enhanced postoperative follow-up—may help improve long-term outcomes.

## Highlights

Current evidence indicates that female patients may have a higher risk of atrial fibrillation (AF) recurrence after catheter ablation compared to males, although findings vary across studies due to methodological heterogeneity.

The underlying mechanisms are multifactorial, involving sex differences in hormonal regulation (e.g., estrogen decline and FSH elevation), atrial substrate characteristics (e.g., more diffuse low-voltage zones and non-pulmonary vein triggers), and structural/functional remodeling (e.g., greater atrial fibrosis and higher prevalence of HFpEF).

Clinical disparities, including later referral for ablation, higher comorbidity burden, and differential anticoagulation responses, further contribute to the elevated recurrence risk observed in women.

A significant treatment selection bias exists, with women being underrepresented in ablation trials and less likely to undergo the procedure, potentially confounding the interpretation of sex-specific recurrence outcomes.

Future efforts should prioritize the development of sex-sensitive risk stratification, substrate-guided ablation strategies, and the integration of advanced imaging and emerging technologies like pulsed-field ablation to enable more personalized and equitable management.

## Introduction

Atrial fibrillation (AF)is the most common clinical tachyarrhythmia [1–2] and, because of its high prevalence, serious complications, and poor prognosis, constitutes a major public health problem [3–4]. Globally, approximately 33.5 million people have AF, and the lifetime risk for adults is 33.3% [5]. Epidemiological data from China show a prevalence of AF of 0.77%, with disability and mortality rates as high as 0.13% and 8.2%, respectively [6–7]. AF is often complicated by severe clinical events such as heart failure, thromboembolism, and stroke, which significantly reduce patients’ quality of life and place a heavy economic burden on healthcare systems [8].

Catheter ablation (CA) for AF yields significantly better outcomes than antiarrhythmic drugs and rate-control therapy [9–10]; however, high postoperative recurrence rates severely limit its clinical application [11–12]. Therefore, investigating risk factors for post-ablation recurrence is of great clinical importance. Recent studies suggest that the risk of recurrence after AF ablation differs significantly by sex, possibly because of factors such as hormonal levels, electrophysiological characteristics, cardiac remodeling, and clinical management strategies. This article aims to systematically review the latest research progress on this topic to provide a theoretical basis for individualized treatment.

This review is a narrative study and did not follow the methodological framework of a systematic review (e.g., it did not use PRISMA diagram, risk-of-bias assessment, and quantitative synthesis). The literature search covered PubMed, Web of Science, China National Knowledge Infrastructure (CNKI), and the Wanfang Data Knowledge Service Platform, primarily for the period from January 2010 to December 2025. In addition, a small number of important studies published between 2005 and 2009 were included by tracking references to trace the origins of key concepts. Search keywords included “atrial fibrillation,” “catheter ablation,” “sex differences,” and “recurrence,” and combinations thereof. Inclusion criteria were: (1) clinical studies (randomized controlled trials, cohort studies, registry studies, etc.) examining sex differences in the risk of recurrence after AF catheter ablation; (2) basic or clinical studies investigating related mechanisms (electrophysiological, structural, hormonal, etc.); and (3) high-quality reviews or commentary articles. Case reports, conference abstracts, and non-Chinese/English language publications were excluded. Two authors independently screened the literature by title, abstract, and full text.

The 2024 European Society of Cardiology (ESC) guidelines for the management of AF propose an integrated management pathway centered on “AF-CARE,” emphasizing that AF is a chronic disease requiring dynamic assessment and multidimensional intervention [1]. The guidelines note that phenotyping should go beyond the traditional paroxysmal/persistent classification to include atrial substrate status, comorbidity profile, risk factors, and psychosocial characteristics. A separate section discusses sex differences: female patients are older at diagnosis, more often have heart failure with preserved ejection fraction (HFpEF), and, despite having more severe symptoms, undergo catheter ablation at a significantly lower rate than men.

This framework provides an important background for the present review: the higher post-ablation recurrence rate in women may not be attributable to sex per se but rather reflects systematic differences in atrial cardiomyopathy phenotype, comorbidity burden, and access to care. These differences correspond precisely to the core intervention components of the AF-CARE pathway: *C* (comorbidity and risk factor management) and E (evaluation and dynamic re-assessment). Future efforts should develop individualized ablation strategies and perioperative management plans based on sex-specific phenotypes to improve outcomes in women and reduce overall recurrence rates.

## Current research on sex differences in AF recurrence risk after CA

Existing studies on the effect of sex on post-ablation AF recurrence rates have not reached consistent conclusions [13]. Several large observational studies suggest that female patients may have a higher risk of recurrence. For example, a cohort study of 5,010 patients showed that the 3-year cumulative recurrence rate was significantly higher in women than in men (43.3% vs. 39.0%, *P* = 0.0046), and multivariable analysis confirmed female sex as an independent risk factor for recurrence (hazard ratio [HR] = 1.24, *P* < 0.0001) [14]. Another registry study from Japan reported that women underwent catheter ablation at a significantly lower rate than men (odds ratio [OR] = 0.608, *P* < 0.0001) [15], reflecting a possible treatment selection bias in clinical practice that may distort the assessment of true recurrence risk in women. However, contradictory evidence also exists. A European multicenter prospective registry study (*n* = 3,593) and a single-center retrospective analysis (*n* = 1,346) both found no significant sex difference in the 1-year post-ablation recurrence rate (34.4% vs. 34.2%, *P* = 0.897; 30% vs. 27.7%, *P* = 0.38) [16–17]. These conflicting results reflect the complexity of the evidence in this area and suggest that our understanding of this issue is still evolving.

When interpreting these heterogeneous studies and attempting to discern overall trends, great caution is required. These studies differ markedly in several key methodological dimensions, including the proportion of AF subtypes (paroxysmal vs. persistent), the scope and technical details of the ablation strategy, the duration of follow-up and intensity of rhythm monitoring, and—critically—the definition of “AF recurrence” itself. These fundamental inconsistencies severely limit the direct comparability of results across studies. Therefore, any general conclusions drawn from such mixed evidence must be viewed as inherently limited, and no firm, unified conclusion can yet be reached. For example, if monitoring is insensitive to asymptomatic recurrences, the true recurrence rate in women may be systematically underestimated, introducing bias into sex comparisons. A systematic analysis of these methodological limitations is presented in Sect. "Methodological limitations and heterogeneity of evidence in existing studies".

In summary, although the available evidence tends to suggest that women may have a higher risk of AF recurrence after catheter ablation, this observation must be interpreted with caution in light of the marked methodological heterogeneity described above. The inconsistency in current findings likely arises from differences in study design, population characteristics, and endpoint assessment. Therefore, future studies that are prospective, rigorously designed, and use uniform endpoint definitions are needed to further test whether sex is independently associated with recurrence risk. At present, the possibility that the observed sex differences are entirely explained by inadequately adjusted confounders cannot be ruled out. Nevertheless, exploring potential biological mechanisms remains scientifically important. To systematically present the core features and results of the key studies meeting the inclusion criteria, we have summarized them in Table 1. As shown in the table, the studies differ considerably in sample size, AF type distribution, follow-up duration, intensity of rhythm monitoring, and recurrence definition; these sources of methodological heterogeneity are likely an important cause of the inconsistent findings.

**Table 1:** Sex differences in atrial fibrillation recurrence after catheter ablation

First author	Year	Country	Sample size	AF type	Follow-up(months)	Monitoring method & recurrence definition	Outcome type	Recurrence rate (M vs. F)	Statistical significance
Yu	2018	Korea	837	EAF	24.5 ± 18.9 (mean ± SD)	ECG/Holter; AF/AT ≥ 30s (3-month blanking)	Arrhythmia recurrence	27.0% vs. 39.0%	*P* < 0.001
Roh	2018	Korea	367	PAF、PersAF	55.0 ± 38.0 (mean ± SD)	ECG, Holter, event recorder; AF/AT > 30s after 3-month blanking	Arrhythmia recurrence	24% vs. 29%	NS(*P* = 0.131)
Grecu	2019	Europe	3593	PAF、PersAF、LSPAF	12.0(median)	ECG/symptom follow-up; AF/AFL ≥ 30s (3-month blanking)	Arrhythmia recurrence	34.2% vs. 34.4%	NS(*P* = 0.879)
Tanaka	2020	Japan	5010	PAF、PersAF、LSPAF	35.0(median)	ECG/Holter; AF ≥ 30s (3-month blanking)	Arrhythmia recurrence	39.0% vs. 43.3%	*P* = 0.005
Li	2022	China	106	PersAF	24.4(median)	12-lead ECG or 24-h Holter; >30s after 3 m blanking	Clinical recurrence	26.0% vs. 55.2%	*P* = 0.005
Jackson	2023	USA	95,394	Mixed AF	12.0(median)	Claims data (no rhythm monitoring); proxy: AF/AFL-related healthcare use	Healthcare utilization	17.0% vs. 17.3%	NS(*P* = 0.38)
Yadav	2023	USA	1346	PAF、PersAF	12.0(median)	ECG, ambulatory monitoring, device data; AF/AT/AFL > 30s post- blanking	Arrhythmia recurrence	27.7% vs. 30.0%	NS(*P* = 0.38)
Ma	2023	China	352	PAF	Not specified	ECG or 24-h Holter; >30 s after 90d blanking	Clinical recurrence	25.6% vs. 32.4%	NS(*P* = 0.191)
Segan	2024	Multinational	338	PersAF	12.0(median)	Device/KardiaMobile/Holter; arrhythmia > 30s off-AADs	Arrhythmia recurrence, AF burden, QoL	43.2% vs. 55.7%	*P* = 0.036
Duarte	2025	Portugal	560	PAF、PersAF	19.0(median)	ECG/Holter/device; AF/AT ≥ 30s post-blanking	Arrhythmia recurrence	HR = 1.73	*P* = 0.033

EAF=Early-onset AF, PAF=Paroxysmal AF, PersAF=Persistent AF, LSPAF=Long-standing persistent AF, Mixed AF = AF cohort identified from healthcare claims data, comprising paroxysmal, persistent, and other unclassified AF types without further subclassification, ECG=Electrocardiogram, Holter = 24-hour Holter monitoring, AT=Atrial Tachycardia, AFL=Atrial Flutter, AADs=Antiarrhythmic Drugs, QoL=Quality of Life, HR=Hazard Ratio,3-month blanking period (unless otherwise specified), Outcome definitions: “M% vs F%” denotes unadjusted absolute recurrence rates; “HR = XX” indicates adjusted hazard ratios from multivariable analysis, NS = Not Significant

## Mechanisms of sex differences in AF recurrence risk after CA

**Fig. 1:**
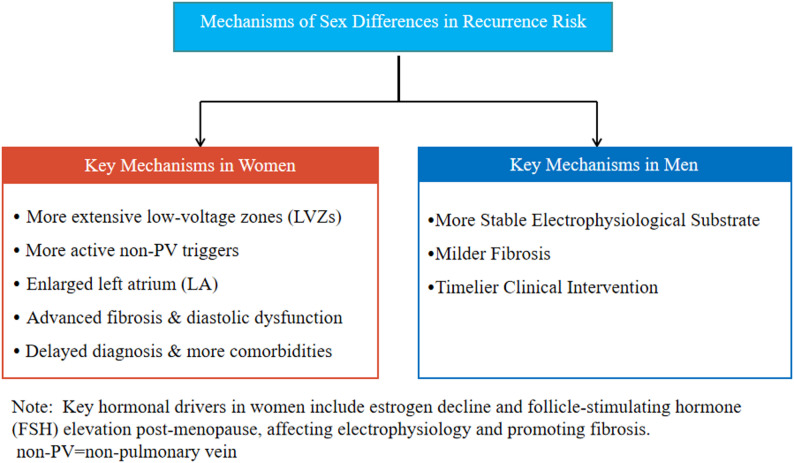
Mechanisms of sex differences in atrial fibrillation recurrence after catheter ablation. Key drivers in women include estrogen decline and FSH elevation after menopause, which affect cardiac electrophysiology and promote fibrosis. Additional female-specific factors include more extensive low-voltage zones (LVZs), more active non-pulmonary vein (non-PV) triggers, enlarged left atrium (LA), advanced fibrosis and diastolic dysfunction, as well as delayed diagnosis and more comorbidities. In men, the contributing factors include more stable electrophysiological substrate, milder fibrosis, and timelier clinical intervention.non-PV, non-pulmonary vein; LVZ, low-voltage zone; LA, left atrium; FSH, follicle-stimulating hormone

Sex differences are not driven by a single factor but arise from the complex interaction of multiple dimensions, including hormonal levels, atrial substrate characteristics, and clinical management strategies. Figure 1 systematically summarizes the main mechanistic pathways and their interrelationships as revealed by current research.

### Hormonal differences

Available evidence suggests that hormonal differences may be one important mechanism mediating sex differences in the risk of recurrence after catheter ablation for AF, potentially acting through multiple pathways including electrophysiological regulation, structural remodeling, autonomic nervous system function, and oxidative stress. At the electrophysiological level, estrogen exhibits bidirectional regulatory effects: studies have observed that it can both inhibit the inward rectifier potassium current (IK1) and the delayed rectifier potassium current (IKr), prolonging the action potential duration and the QT interval, and upregulate the L-type calcium current (ICa, L), increasing intracellular calcium concentration. The latter can induce calcium overload and delayed afterdepolarizations, thereby increasing arrhythmogenic risk [18–19]. Regarding structural remodeling, physiological concentrations of estrogen inhibit pro-fibrotic signaling pathways such as transforming growth factor-beta (TGF-β)/Smad, reducing collagen deposition. However, after menopause, estrogen levels decline markedly, accompanied by a relative increase in progesterone activity, which may abnormally activate fibrotic pathways and promote extracellular matrix remodeling; this may increase the risk of post-ablation recurrence [19–21]. In addition, autonomic regulation and oxidative stress pathways may also be influenced by hormones [19–22]. Postmenopausal estrogen/progesterone imbalance can enhance sympathetic tone, increasing the occurrence of ectopic rhythms through activation of β-adrenergic receptors. Concurrently, hormonal imbalance can activate the nuclear factor kappa-B (NF-κB) pathway and lead to accumulation of reactive oxygen species (ROS), forming a vicious cycle of “inflammation–oxidative stress–electrical remodeling.”

In recent years, the mechanism of follicle-stimulating hormone (FSH)-mediated oxidative damage has attracted attention. A clinical study suggested that elevated FSH levels in postmenopausal women correlate positively with the area of low voltage in the left atrium. Animal experiments further found that FSH can induce mitochondrial dysfunction, alter the expression of oxidative stress-related genes, maintain a state of elevated ROS, and may directly damage cardiomyocytes and activate fibrotic signaling. In models of spontaneous AF and pressure overload, an FSH blocker reversed these pathological processes [23]. It should be emphasized that these findings come primarily from clinical observational associations and preclinical animal experiments; the role of FSH as a therapeutic target and its clinical significance in women with AF remain to be validated in prospective studies.

Existing observational studies have found that the mean age of women undergoing catheter ablation is significantly higher than that of men, and most women are postmenopausal [16]. This observation suggests that menopause may be an important node for understanding sex differences: postmenopausal women often show more extensive low-voltage zones in the atria and a greater fibrotic burden than premenopausal or perimenopausal women, and these indices are statistically associated with their higher post-ablation recurrence risk. However, whether this association reflects a causal relationship or is due to other accompanying factors such as increasing age or longer disease duration remains uncertain. The decline in estrogen and the compensatory rise in FSH may act synergistically through mechanisms such as promoting oxidative stress, enhancing fibrotic signaling, and altering autonomic tone to jointly exacerbate the progression of atrial myopathy. Therefore, when evaluating hormone-related sex differences, it is necessary to include age and menopausal status as important stratification factors in the analytical framework to more accurately explore the underlying pathophysiological connections. Ultimately, hormonal changes influence ablation outcomes through downstream structural and functional remodeling of the atria. The next section further elaborates on the central role of fibrosis, electrophysiological abnormalities, and functional changes in sex differences from the integrated perspective of atrial myopathy.

### Atrial myopathy: an integrated framework of structure, electrophysiology, and function

#### Structural–electrophysiological–functional differences

Atrial myopathy has been proposed as a potential integrated pathological framework for understanding sex differences in outcomes after catheter ablation for AF. Available evidence suggests that female patients may show systematic worsening across multiple dimensions, including fibrosis, electrophysiological abnormalities, diastolic dysfunction, and left atrial enlargement. The core of structural remodeling is atrial fibrosis, and women often present with a more extensive and diffuse pattern of fibrosis that directly disrupts the homogeneity of electrical conduction and provides a substrate for re-entry [20, 24–27]. Functionally, HFpEF is particularly common in women and leads to chronic elevation of left atrial afterload through left ventricular diastolic dysfunction, accelerating the progression of fibrosis [28–30]. At the electrophysiological level, diffuse fibrosis manifests as widely distributed, poorly demarcated low-voltage zones. These dimensions interact with one another—structure, electrophysiology, and function—forming a self-reinforcing vicious cycle that may partly explain the higher post-ablation recurrence rate in women.

#### Technical limitations of voltage mapping

The use of voltage mapping to assess the atrial substrate has inherent limitations that are particularly pronounced in the diffuse low-voltage zones typical of women: insufficient spatial resolution may underestimate diffuse disease, the lack of standardized voltage thresholds compromises comparability of results, and voltage signals are susceptible to interference from catheter contact force, local edema, and autonomic fluctuations [25–27]. This means that relying solely on conventional voltage mapping cannot accurately depict the full extent of the atrial substrate in women and may systematically bias the mechanistic interpretation of sex differences.

#### Clinical challenges for ablation strategies

These distinctive pathological features directly translate into challenges for intervention: non-pulmonary vein (non-PV) triggers are more common and more diffusely distributed in women, limiting the efficacy of ablation strategies centered on pulmonary vein isolation (PVI) [31–33]. Despite the presence of diffuse substrate abnormalities, current evidence does not support routine empirical expansion of the ablation lesion set—adding ablation of low-voltage zones to PVI did not significantly reduce recurrence rates in women [32]. This is likely because diffuse, poorly demarcated fibrotic tissue makes it more difficult to achieve transmural, durable lesions, and because it interacts with disease burdens that are common in women, such as HFpEF, affecting tissue responsiveness and repair [34–36]. In this context, expanded ablation strategies aimed at modifying the arrhythmogenic substrate have received considerable attention. However, DECAAF II, a large-scale randomized controlled trial (RCT), showed that adding late gadolinium enhancement cardiac magnetic resonance (LGE-CMR)-guided fibrosis-targeted ablation to PVI did not reduce the risk of recurrence in patients with persistent AF and instead increased perioperative stroke; moreover, women accounted for only 21.1% of the study population, and sex-specific outcomes were not reported [37]. These findings suggest that a “one-size-fits-all” substrate modification strategy for diffuse fibrosis may be ineffective or even harmful, and future efforts should focus on accurately identifying sex-specific phenotypes (e.g., focal low-voltage zones) that would truly benefit.

#### Key confounding factors

When dissecting the above mechanisms, two key confounding factors must be carefully considered. First is the clinical phenotype of AF: the proportion of persistent AF may be higher in women, and persistent AF itself is associated with more extensive low-voltage zones, more severe structural remodeling, and a higher proportion of non-PV triggers. If AF type is not adequately adjusted for, the risk associated with “persistent AF” may be erroneously attributed to “female” sex [33]. Second is body size difference: men typically have larger absolute cardiac dimensions and body surface area (BSA), which can affect the interpretation of absolute voltage mapping values and may influence ablation efficacy by affecting catheter contact and energy delivery efficiency [32–34]. Future studies should systematically adjust for AF subtype and body size indices to avoid mistakenly attributing modifiable clinical characteristics to biological sex itself.

### Sex differences in clinical management

In line with the mechanistic hypotheses above, sex differences at the level of clinical management also deserve attention. Existing studies show that female patients with AF have systematic differences in clinical characteristics, treatment patterns, and outcomes, and these differences collectively influence their risk and decision-making regarding catheter ablation. Multiple large-scale epidemiological studies have demonstrated that women experience significant delays in diagnosis and treatment, with a mean referral time approximately 1.5 years later than men. This means that when they present for care, their disease is often at a more advanced stage, characterized by a higher proportion of non-paroxysmal AF, more extensive atrial fibrosis, and more non-PV triggers, all of which can increase the risk of post-ablation recurrence [38–40].

Sex differences are also pronounced in treatment delivery and complications [28, 40]. The proportion of women undergoing catheter ablation is significantly lower than that of men, yet their rate of procedure-related complications is higher, particularly for vascular injury, pericardial effusion, pericarditis, and bleeding events. This may be related to sex-specific anatomical and physiological characteristics, such as relatively slender femoral vessels and thinner atrial walls, which make vascular access and catheter manipulation more challenging. Anticoagulation strategies also differ by sex: although some data show that anticoagulation rates may be higher in women, meta-analyses indicate that even with direct oral anticoagulants (DOACs), the risk of stroke and systemic embolism remains significantly higher in women than in men, while the risk of major bleeding is relatively lower. This unique balance of thrombotic and bleeding risk needs to be considered in perioperative management [41–42].

In addition, sex differences are also evident in comorbidity management and functional status. Epidemiological data show that women present at an older age and more often have comorbidities such as hypertension, valvular heart disease, diabetes, hyperthyroidism, and HFpEF, whereas coronary artery disease and heart failure with reduced ejection fraction (HFrEF) are more common in men [43–45]. Obesity and frailty are more prevalent in women, especially postmenopausal women. Obesity can promote atrial remodeling through pro-inflammatory states and hemodynamic changes; frailty may affect surgical tolerance and postoperative recovery [39–41]. At the same time, there are sex inequalities in the accessibility and quality of clinical services. Women may not receive adequate management of left atrial structural assessment, blood pressure control, or rhythm therapy, and the underrepresentation of women in RCTs limits the generalizability of evidence [44–47].

In summary, decisions about ablation for female patients with AF need to be based on a deep understanding of their unique clinical phenotype, increased risk of complications, and management of complex comorbidities. The “AF-CARE” pathway proposed by the 2024 ESC guidelines for AF management—C comorbidity and risk factor management, A avoidance of stroke and thromboembolism, R symptom reduction with rate and rhythm control, and E evaluation and dynamic re-assessment—provides an actionable framework for integrating these sex-sensitive factors [1]. The guidelines explicitly state that when assessing suitability for ablation, comorbidity management (e.g., HFpEF, obesity), risk factor control, and patient participation should be systematically incorporated, rather than using sex alone as a decision-making criterion. The guidelines also emphasize that a phenotyping strategy—that is, moving beyond the label of “female” to quantify specific atrial fibrosis burden, distribution of non-PV triggers, and comorbidity severity—is a prerequisite for optimizing individualized treatment. On this basis, one could attempt to develop sex-sensitive clinical pathways that align with the AF-CARE components: early identification and intervention correspond to the C component; optimizing patient selection and improving procedural techniques (e.g., ultrasound-guided puncture) correspond to the R component; and individualized anticoagulation management and enhanced multidimensional assessment (including metabolic and functional status) correspond to the A and E components. However, whether these strategies can control risk while improving treatment adherence and long-term efficacy, and ultimately improve overall prognosis in women, is not yet supported by direct prospective evidence and awaits future validation. With respect to early rhythm control, the EAST-AFNET 4 trial demonstrated that early rhythm control significantly reduced the composite endpoint of cardiovascular death, stroke, and hospitalization for heart failure compared with usual care [48]. A post-hoc analysis of that trial further showed that this benefit was consistent across different AF types (newly diagnosed, paroxysmal, persistent), but patients with newly diagnosed AF in the early rhythm control group had a higher risk of hospitalization for acute coronary syndrome and more days in hospital. However, that trial also lacked pre-specified sex subgroup analyses. Given that women often have a longer disease duration and more advanced atrial remodeling at presentation, their absolute benefit from early rhythm control may differ from that of men, but direct evidence for this is currently lacking.

## Methodological limitations and heterogeneity of evidence in existing studies

Studies of sex differences in the risk of recurrence after catheter ablation for AF show marked inconsistencies in their conclusions. The root cause is profound heterogeneity across multiple dimensions, including study design, population composition, clinical characteristics, assessment methods, and geographic context. This heterogeneity is widely evident in key aspects such as the source of evidence, selection of study populations, definition of endpoints, and methods of assessment, and it is further modulated by differences in healthcare systems and sociocultural factors across regions, all of which contribute to the inconsistent findings on sex differences between cohorts.

Heterogeneity in the source of evidence and in study populations first weakens comparability between studies. The underrepresentation of women in RCTs (median proportion 31.5%) limits their generalizability [37]; in key trials such as EAST-AFNET 4, DECAAF II, and ADVENT, the proportions of women were 46.3%, 21.1%, and approximately 33%, respectively, and none had pre-specified sex subgroup analyses or adequate statistical power [43, 48–49]. Observational studies are often subject to selection bias and unmeasured confounding. Study populations differ significantly in AF type distribution, age distribution, and comorbidity burden, and these factors themselves show sex-specific characteristics: female patients typically present at an older age, have a higher proportion of persistent AF, and have a greater burden of atrial myopathy. Failure to adequately adjust for these baseline differences can lead to the erroneous attribution of group characteristics to sex itself. AF subtype is a key source of heterogeneity: women have a higher proportion of persistent AF, which is often accompanied by more pronounced atrial remodeling. Age and menopausal status are also important confounders, but most studies lack stratified analyses for these factors. Furthermore, whether precise imaging techniques such as LGE-CMR are used to quantify atrial fibrosis burden directly affects the ability to adjust for confounders. Studies that do not adequately assess the substrate tend to attribute the higher recurrence rate in women simply to sex, whereas the independent effect of sex often diminishes markedly after adjusting for substrate burden, suggesting that the burden of atrial myopathy may be a more central driver [44–45, 50]. The widespread delay in diagnosis and treatment in women means that they often undergo ablation at a more advanced stage of disease. Differences in referral patterns across healthcare systems (e.g., a more mature healthcare system in the United States may shorten the time from diagnosis to intervention) further complicate cross-regional comparisons of sex differences and may partly explain why sex differences appear smaller in US studies than in those from other countries [17, 50].

To test the hypothesis that “female sex may primarily act as a proxy variable for confounders such as atrial myopathy, comorbidities, and AF subtype,” this review attempted to extract trends in the change of the risk ratio for female sex before and after multivariable adjustment from the original studies. Tanaka et al., using the KPAF registry (*n* = 5,010), reported that female sex remained an independent predictor after adjustment for 19 covariates (HR = 1.24, 95% confidence interval [CI]: 1.12–1.38, *P* < 0.0001), but that study did not include key substrate variables such as atrial fibrosis burden and HFpEF [14]. Notably, in that study, the proportion of non-paroxysmal AF and left atrial diameter were both significantly lower in women than in men, yet the recurrence rate was higher in women, and women underwent more ablation of non-PV foci (4.2% vs. 2.6%) and superior vena cava isolation (15.7% vs. 12.8%), suggesting that non-traditional risk factors may play an important role. Yu et al., in a study of early-onset AF (< 60 years) (*n* = 1,060), found that female sex remained independently associated with a higher risk of recurrence after multivariable adjustment (HR = 2.58, 95% CI: 1.06–6.30, *P* = 0.037), and women had worse baseline substrate indices, including left atrial voltage, diastolic function (E/Em), and atrial volume index [22]. However, that study did not use a stepwise adjustment strategy, so it is not possible to directly quantify the degree to which the effect of sex attenuated after inclusion of substrate variables. Combining these two studies, when adjustment models include traditional clinical variables, the independent effect of female sex remains significant (HR 1.24–2.58), but the systematic disadvantage of women at the substrate level may constitute the pathological basis for the higher recurrence rate. Because no study has reported stepwise changes in HR with sequential adjustment in the same dataset, the precise extent of attenuation of the sex effect cannot be quantified. In both of these studies, the proportion of non-paroxysmal AF was lower in women than in men, yet the recurrence rate was higher in women; this counterintuitive finding further supports the hypothesis that “female sex may proxy for non-traditional risk factors such as diffuse fibrosis, non-PV triggers, and HFpEF,” but this hypothesis remains inferential and needs to be tested in prospective studies with a pre-specified stepwise adjustment strategy.

Critical methodological heterogeneity further complicates sex comparisons, particularly in the substantial inconsistency in the definition of the core endpoint “AF recurrence” [30, 44, 50]. There is no uniform definition of the post-ablation “blanking period,” and differences in its definition may confound estimates of long-term recurrence rates because of sex differences in the response to early electrical remodeling. Rhythm monitoring strategies vary enormously in sensitivity and duration, ranging from symptom-dependent intermittent electrocardiogram (ECG) recording to long-term implantable loop recorders, with differing abilities to detect asymptomatic or paroxysmal AF. Because the proportion of asymptomatic recurrences may be higher in women, monitoring strategies with insufficient sensitivity will systematically underestimate their recurrence rate, introducing bias into sex comparisons. The definition of recurrence is also highly heterogeneous with respect to the type of arrhythmia, the duration threshold, and the method of counting, which may have bidirectional effects on estimates of sex differences. Moreover, most studies have not systematically adjusted for body size-related variables (e.g., BSA, body mass index [BMI]), which can affect atrial dimensions, voltage mapping thresholds, and the extent of ablation lesions; failure to adjust for these can confound sex comparisons of electrophysiological parameters and outcomes. The use of perioperative and post-ablation antiarrhythmic drugs lacks standardization and is often not reported by sex; maintenance of sinus rhythm with drugs may mask true recurrence risk. Statistical analysis methods also have limitations. Most observational studies do not adequately adjust for key variables such as the extent of atrial fibrosis, specific ablation details, body size indices, and socioeconomic factors, leading to residual confounding. In addition, subgroup analyses within sexes often have low statistical power because of small sample sizes [46–47, 50].

In summary, the core challenge in interpreting sex differences in AF recurrence after catheter ablation is to distinguish between “sex” as an independent biological predictor and its role as a proxy for a series of clinical and social factors. The higher recurrence rate observed in women in many current studies may primarily reflect the more advanced stage at presentation, greater burden of atrial myopathy, specific comorbidity profile, and referral delays that are more common in this population. Some studies suggest that when study designs systematically and precisely adjust for the factors driving these “apparent differences”—particularly by quantifying atrial fibrosis with imaging—the effect of sex itself as an independent risk factor is often attenuated [25–26]. Therefore, future studies should re-evaluate the independent contribution of sex only after adequately adjusting for these confounders, so as to avoid erroneously attributing modifiable clinical management gaps or specific pathophysiological phenotypes to biological sex itself. In addition, when interpreting studies from different geographic origins, the potential moderating effects of multiple sociocultural factors—including the level of development of the healthcare system, population genetics and comorbidity characteristics, standards of surgical technique, follow-up monitoring strategies, and access to health insurance—need to be carefully considered. Future efforts should promote prospective studies that include more representative female populations, develop and adhere to uniform definitions for recurrence monitoring and endpoints, and systematically adjust for multidimensional confounders, including clinical, imaging, procedural, and socioeconomic variables. At present, the translation of evidence into clinical practice in this area still faces significant knowledge gaps.

## Summary and future directions

Current evidence cannot completely rule out the possibility that female sex itself is an independent biological predictor, but an increasing number of studies suggest that the observed higher recurrence risk may primarily reflect the effects of confounders such as later presentation, greater burden of atrial myopathy, a unique comorbidity profile, and a distinct metabolic–hormonal environment. On this basis, some authors have proposed a hypothetical framework: sex differences in recurrence are essentially a reflection of baseline risk differences and diagnostic–therapeutic delay, and “female sex” may act as a “proxy variable” for these complex clinical characteristics. It should be emphasized that this explanatory framework is still hypothetical, and its validity needs to be rigorously tested in prospective studies that systematically adjust for the above confounders.

Future research should move beyond simple binary sex comparisons and aim to construct an individualized management framework that spans the entire diagnostic and therapeutic continuum. One potential approach is to develop a sex-sensitive risk stratification system, with a particular focus on postmenopausal women and those with HFpEF or left atrial enlargement. Improving the recognition of atypical symptoms and enhancing rhythm monitoring may shorten diagnostic and therapeutic delays, interrupting the “delay–remodeling–recurrence” chain. Preoperative use of LGE-CMR to quantify atrial fibrosis burden, combined with intraoperative high-density voltage mapping, can provide a basis for individualized ablation. The AF-CARE pathway and the concept of phenotyping proposed in the 2024 ESC guidelines should serve as a methodological cornerstone for future research. Specific directions include: ① Phenotyping: construct sex-specific phenotypic maps based on a multidimensional framework that includes atrial substrate status, comorbidity profile, risk factors, and psychosocial characteristics. ② Integrated management: prospectively validate the inclusion of variables such as hormonal status, atrial fibrosis burden, HFpEF, and diagnostic delay into the C (comorbidity management) and E (dynamic evaluation) components of AF-CARE. ③ Dynamic assessment: sex differences may present different patterns at different stages, such as between first and repeat ablation or between premenopausal and postmenopausal status, necessitating multiple time-point assessments.

The primary task is to promote the construction of a “phenotypic map of female atrial myopathy” through multicenter studies that systematically characterize combinations of different fibrosis patterns, trigger distributions, and electrophysiological properties to identify clinically meaningful subtypes. In terms of treatment strategy, while ensuring PVI, targeted substrate modification may be considered in patients with a clearly defined arrhythmogenic substrate or non-PV triggers, avoiding indiscriminate expansion of the ablation lesion set. In response to the more diffuse fibrosis and thinner atrial tissue that are more common in women, the development of “substrate-adaptive ablation” parameters should be explored. Pulsed-field ablation (PFA) has the potential advantages of tissue selectivity and avoidance of thermal injury, which may theoretically be more beneficial for women, who are more likely to have thinner atrial tissue and more comorbidities; however, sex-specific efficacy and safety data for PFA in women are currently lacking.

Differentiated perioperative and long-term management pathways need to be explored, including the identification, prevention, and management of sex-specific complication risks. In addition to exploring the potential role of sodium-glucose cotransporter 2 (SGLT2) inhibitors and angiotensin receptor-neprilysin inhibitors (ARNIs) in women with specific heart failure phenotypes, the feasibility of “hormonal status-guided perioperative management” could be investigated, for example, by examining the value of short-term hormonal modulation in postmenopausal women to reduce post-ablation inflammation and fibrotic activity. During follow-up, more intensive monitoring strategies should be used to increase the detection rate of asymptomatic recurrences, allowing dynamic optimization of treatment.

In summary, one potential path toward more individualized treatment and management lies in developing an integrated assessment system that systematically incorporates and adjusts for key confounders such as menopausal status, AF subtype, atrial structure and extent of fibrosis, obesity, and frailty. In theory, such a refined analytical strategy could help to identify potentially modifiable pathophysiological mechanisms and clinical intervention points from the observed sex differences. However, the clinical feasibility and effectiveness of this framework still need to be validated in prospective interventional studies. Until then, we should be cautious about directly translating hypotheses that are still at the conceptual stage into clinical recommendations. Only through such a refined, de-superficialized research strategy will it be possible to extract truly modifiable pathophysiological mechanisms and clinical intervention points from the observed sex differences, thereby exploring more precise and effective long-term management pathways for all patients with AF.

## Data Availability

No datasets were generated or analysed during the current study.
